# Psychometric validation of the Czech PLOC-R in high-school physical education

**DOI:** 10.3389/fspor.2025.1629138

**Published:** 2025-09-30

**Authors:** Ivana Harbichová, Jan Štochl, Lawrence M. Scheier, Martin Komarc

**Affiliations:** ^1^ Department of Methodology, Faculty of Physical Education and Sport, Charles University, Prague, Czechia; ^2^ The Psychometrics Centre, Department of Psychiatry, University of Cambridge, Cambridge, United Kingdom; ^3^ LARS Research Institute, Inc., Sun City, AZ, United States; ^4^ Prevention Strategies, Greensboro, NC, United States

**Keywords:** motivation, self-determination theory, PLOC, physical education, psychometric validation

## Abstract

**Introduction:**

The Perceived Locus of Causality (PLOC) scale is widely used to assess motivation in physical education (PE), but no validated Czech version has been available. This study aimed to translate, adapt, and validate the revised PLOC (PLOC-R) in Czech high school students.

**Methods:**

A total of 2,967 students (mean age = 16.62, SD = 1.18) completed the Czech-adapted PLOC-R along with measures of psychological need satisfaction and PE engagement. Confirmatory factor analysis (CFA) tested its five-factor structure, while exploratory analyses identified problematic items.

**Results:**

The initial model showed moderate fit, but removing three items resulted in better factorial validity of the scale (CFI = 0.94, RMSEA = 0.06). Internal consistency was acceptable (*ω* = 0.78–0.90), except for external regulation (*ω* = 0.58). The scale demonstrated strict measurement invariance across gender and grade, and correlations with external variables supported its validity.

**Discussion:**

This study provides the first validated Czech PLOC-R, enabling reliable assessment of PE motivation and facilitating cross-cultural comparisons.

## Introduction

The benefits of physical activity (PA) have been widely demonstrated in the last decade (1). High school physical education (PE) plays a major role in delivering these benefits and since it is the last form of mandatory PA for students, PE also serves as the final opportunity for broad, institutionally supported promotion of PA among children and adolescents (2). However, this role of school-based PE can only be fully endorsed if pupils experience PE positively and therefore an important component to understanding the process of maintaining high interest in PE necessitates a better grasp of the motivational processes that inspire youth to take part in school PE.

One of the most widely accepted frameworks for understanding motivation in PE is Self-Determination Theory (SDT), a comprehensive theory of human motivation that distinguishes between types of motivation based on the degree of self-determination, or autonomy, involved. Developed by Deci and Ryan (3), SDT proposes that motivation exists on a continuum, ranging from amotivation (a lack of motivation) through varying forms of extrinsic motivation, to intrinsic motivation. This continuum reflects the extent to which individuals feel self-directed vs. controlled in their actions, which has critical implications for engagement, persistence, and the psychological benefits derived from participation. Central to SDT is the concept of basic psychological needs satisfaction, specifically the needs for autonomy, competence, and relatedness (4). When these needs are adequately met, individuals are more likely to experience more self-determined (autonomous) motivation and, consequently, enhanced enjoyment, persistence, and well-being in PE (5).

The SDT has been widely applied to PE settings, where fostering autonomous motivation is seen as crucial for encouraging long-term participation in PA. Indeed, research in PE classrooms shows that when students feel more self-driven in PE, they exhibit greater enjoyment, effort, and commitment, both in class and in related out-of-school PA (6). In contrast, more controlling motives (e.g., purely external demands) tend to be associated with lower participation and negative attitudes (4). These findings underscore the importance of understanding what drives student motivation in PE, as it can inform teaching strategies that support the basic psychological needs of autonomy, competence, and relatedness—conditions that nurture more self-determined motivation (4). To effectively measure motivation in PE and understand the diversity of students' motivational orientations, it is essential to use reliable, theoretically sound and contextually appropriate instruments. Given the absence of such an instrument in Czechia, this study aims to adapt and validate the Czech version of the revised Perceived Locus Of Causality (PLOC-R) scale (7), one of the most prominent motivation measures worldwide.

### The perceived locus of causality (PLOC) scale and its revised version

Both the PLOC and PLOC-R scales, grounded in SDT, are widely used to assess different motivational types along the self-determination continuum. The PLOC scale was designed to capture the extent to which students perceive their reasons for engaging in PE as self-determined/autonomous vs. controlled or amotivated. The PLOC was originally developed by Goudas et al. (8) as a PE-specific adaptation of existing motivation questionnaires. It was created by modifying items from Ryan and Connell's (9) academic Self-Regulation Questionnaire to represent external, introjected, identified, and intrinsic regulations (4 items each), and by incorporating an amotivation subscale (3 items) derived from Vallerand et al.'s (10) Academic Motivation Scale. Goudas et al. (7) reported acceptable internal consistency (Cronbach's *α* > .70) for all subscales in initial validation tests. Notably, integrated regulation was not included in the adolescent-focused PLOC, consistent with the notion that this fully internalized form of extrinsic motivation is more common in adults.

Over the past decades, the PLOC has become a widely used instrument for assessing motivation in school PE and has been instrumental for research in PE settings (11). Studies using the PLOC have supported its validity, showing that students' responses generally follow the expected simplex pattern (i.e., subscales array along the self-determination continuum, with adjacent motives more positively correlated than distant ones) and correlate meaningfully with educational outcomes (12). At the same time, a few studies have elucidated psychometric challenges with the original PLOC. For example, very high correlations between certain subscales (such as identified and introjected regulation) have been reported, raising questions about their discriminant validity (13). In response to such issues, a revised PLOC (PLOC-R) scale was later developed by Vlachopoulos et al. (7), refining item wording and scale composition.

### Cross-cultural validation of the PLOC and PLOC-R

Because student motivation is shaped by both cultural and educational contexts, the Perceived Locus of Causality Questionnaire (PLOC) and its revised version (PLOC-R) have undergone numerous validations across countries and populations. While research has generally confirmed the five-factor structure (amotivation, external, introjected, identified, and intrinsic regulation) and internal consistency of these instruments, findings have also highlighted the importance of context-specific validation when adapting the tool for new cultural or linguistic settings.

In Spain, Trigueros et al. (14) validated the PLOC-R among more than 1,900 secondary students, confirming the five-factor model and reporting adequate fit indices and reliability (Cronbach's *α* > .70 for most subscales). Wang et al. (12) examined cross-cultural validity between British and Singaporean students, demonstrating configural and metric invariance across individualistic and collectivist cultural contexts. They also found that British students reported higher levels of intrinsic motivation and lower levels of controlled regulation compared to their Singaporean peers, underscoring cultural influences on motivational profiles even when structural validity is maintained.

Wolf et al. (15) extended the scale by adding integrated regulation items to reflect the full SDT continuum. Their validation with German-speaking high school students supported a six-factor, 24-item model and revealed a predominantly simplex-like pattern of inter-factor correlations. Likewise, Teixeira et al. (16) tested the PLOC-R with over 1,300 Portuguese high school students. After removing two problematic items, the final 18-item version showed good model fit (CFI ≈.92; RMSEA ≈.067) and invariance across gender groups. A cross-cultural comparison by Yang et al. (17) revealed that several PLOC-R items did not function equivalently across Chinese and Spanish samples. Removing three non-invariant items yielded a 17-item version with good fit and acceptable validity in both contexts.

In addition, a number of further studies (18–20) have used the PLOC(-R) as part of broader cross-cultural research in PE contexts, often confirming the theoretical structure but also noting the need for modifications. Taken together, this body of work confirms that while the PLOC-R is a psychometrically sound instrument, its adaptation must be empirically tested in each new context to ensure linguistic clarity, cultural relevance, and conceptual equivalence.

### Present study

Despite its widespread use, no validated Czech version of the PLOC-R currently exists. This presents a twofold limitation: researchers in the Czech Republic lack access to a standardized instrument for assessing PE-related motivation, and Czech student data cannot yet be meaningfully integrated into international research using SDT-based frameworks. For practitioners, especially PE teachers, this also means there is no evidence-based tool available to identify different motivational profiles among students, assess the impact of instructional practices, or tailor interventions to foster more self-determined forms of motivation.

Validating the PLOC-R in the Czech educational context is therefore a necessary step. A well-established Czech version will facilitate reliable measurement of student motivation in PE settings, support instructional decision-making, and enable meaningful cross-cultural comparisons in future research.

The aim of this study was to validate the PLOC-R in a Czech sample of high school students. Specifically, we sought to determine whether the five-factor structure of the PLOC-R holds in the Czech language and context, and whether the instrument demonstrates acceptable reliability, validity, and measurement invariance across gender and grade levels. Establishing these properties is essential to ensure that the scale performs well both psychometrically and practically.

We hypothesized that:

a. PLOC-R responses would demonstrate adequate fit within a correlated five-factor CFA model;
b. all subscales would yield McDonald's omega (*ω*) values greater than.70, indicating acceptable internal consistency;
c. the correlations among subscales would follow a simplex-like pattern along the self-determination continuum;
d. the instrument would demonstrate measurement invariance across gender and high school grade levels; and
e. the PLOC-R factor scores would show theoretically consistent correlations with key constructs from the SDT nomological network, such as basic psychological need satisfaction and PE engagement.

## Method

### Participants and procedures

Data for this cross-sectional study were taken from the first wave of a three-year longitudinal project examining the relationship between PE motivation and cognitive functioning among high school students in the Czech Republic. The Czech Republic comprises 14 administrative regions, which were consolidated based on geographical proximity into five contiguous areas for sampling purposes. Schools were randomly selected from each area, with one large school (>450 students) and one or two smaller schools (<450 students) drawn from each. School administrators were individually approached and invited to participate in the longitudinal study. Of those contacted, six schools declined participation, and recruitment concluded after 13 schools agreed to participate, ensuring a relatively even distribution across the five geographic areas.

Participating schools announced the study to students through flyers and homeroom announcements and provided a hyperlink to an online survey. As an incentive, students who completed the survey were entered into a raffle for a gift card, conducted separately at each school. Participation rates varied widely by school, ranging from a minimum of 10% to a maximum of 86.7%, with an average participation rate of 53.7%.

The final sample for the current study comprised *n* = 2,967 students, with a mean age of 16.62 years (SD = 1.18). A detailed description of the sample, including a breakdown by gender and age categories, is presented in Table 1. More than half of the participants were female (52.5%), and the majority (94.3%) identified as Czech nationals. Other ethnic groups included Ukrainian (2.3%), Slovak, Vietnamese, Roma, and other (each representing 1% or less). Approximately two-thirds of participants (63%) reported living in a two-parent household, with an average household size of 3.84 members (SD = 1.10).

**Table 1:** Sample characteristics by age and gender for study demographic measures.

Variable	Female (*n* = 1,558)	Male (*n* = 1,348)	p	15 years (*n* = 565)	16 years (*n* = 910)	17 years (*n* = 717)	18-19 years (*n* = 721)	p
Age, years (M, SD)	16.61 (1.12)	16.63 (1.20)	0.671	–	–	–	–	
Parent education, years (M, SD)	13.1 (2.04)	13.16 (2.08)	0.431	13.06 (2.03)	13.14 (2.08)	13.16 (2.01)	13.17 (2.14)	0.788
Grades (M, SD)	3.04 (1.42)	3.8 (1.56)	**0**.**000**	3.16 (1.41)	3.36 (1.52)	3.44 (1.58)	3.54 (1.56)	**0**.**000**
School absence, hours (M, SD)	47.65 (39.22)	43.99 (40.07)	**0**.**014**	45.89 (44.24)	44.04 (39.38)	44.37 (36.03)	49.92 (39.74)	**0**.**016**
Sex (*N*, %)								**0**.**009**
Female	–	–	–	312 (56.1)	441 (49.8)	408 (57.8)	379 (53.5)	
Male	–	–	–	244 (43.9)	445 (50.2)	298 (42.2)	330 (46.5)	
Nationality Czech (*N*, %)			0.433					0.070
No	78 (5)	76 (6)		42 (7.5)	45 (5)	38 (5.3)	30 (4.2)	
Yes	1,476 (95)	1,263 (94)		521 (92.5)	859 (95)	675 (94.7)	684 (95.8)	
Sport participation (*N*, %)			**0**.**000**					**0**.**043**
None	1,099 (71)	706 (52)		337 (60)	546 (60)	451 (63)	481 (67)	
Recreational/local level competitions	314 (20)	494 (37)		158 (28)	262 (29)	199 (28)	182 (25)	
Inter/national level competitions	145 (9)	148 (11)		70 (12)	102 (11)	67 (9)	58 (8)	
Two-parent household (*N*, %)			0.562					0.149
No	564 (36.2)	502 (37.2)		202 (35.8)	327 (35.9)	250 (34.9)	290 (40.2)	
Yes	994 (63.8)	846 (62.8)		363 (64.2)	583 (64.1)	467 (65.1)	431 (59.8)	

Parent education averages father + mother. *p*-values represent omnibus comparison using the analysis of variance (continuous variables) and *χ*2 test (count variables).

Bold values are significant at 0.05 level of significance.

Significant differences were observed across several demographic indicators. Girls reported better average grades than boys, whereas boys had slightly fewer hours of school absence. Sport participation also differed by gender, with boys more frequently engaged in organized physical activity, particularly at competitive levels. With increasing age, average grades declined and school absence slightly increased. A trend toward lower sport participation in older students was also observed. These patterns align with known developmental trends in adolescent academic and extracurricular engagement and support the representativeness of the sample.

During the consenting procedure (IRB# 142/22 Ethics Committee, FTVS UK), all participants were informed about the purpose of the study and of their ethical rights as research participants. Participation in the anonymous study was completely voluntary, and participants were free to terminate their participation in the study at any time with no resulting penalty.

Instruments not previously validated in Czech were translated from original language (English) using a multistep translation and adaptation process to ensure both linguistic accuracy and cultural appropriateness (21, 22). Initially, three independent forward translations were produced by bilingual professionals with expertise in psychological and educational terminology. These versions were then synthesized through an expert panel discussion involving the translators and one of the study's co-authors, with the aim of achieving semantic and conceptual equivalence. The appropriateness of item formulations for Czech high school students was further reviewed in an in-person consultation with a high-school teacher. The reconciled version was subsequently subjected to cognitive pretesting with a small sample of students (*n* = 6; 3 males, 3 females). During this stage, item-by-item protocol analysis was conducted to assess clarity, interpretability, and potential ambiguities. Based on participant feedback, only minor wording adjustments were made to improve comprehensibility while preserving the theoretical integrity of each item. The finalized Czech version was then used in the subsequent psychometric validation phase.

### Measures

#### SDT-based motivation in PE – PLOC-R scale

The PLOC-R (13) consists of 19 items, with each of the five subscales containing four items—except for the External Regulation subscale, which includes only three. To address this, the research team developed a fourth item for this subscale during the translation and adaptation process. The rationale for this addition was primarily methodological: a three-item factor results in a saturated one-factor confirmatory factor analysis model, which prevents the evaluation of factorial validity due to perfect model fit. Since assessing each subscale's structure independently is a recommended practice in latent variable modeling, the additional item was introduced to allow proper testing of the External Regulation factor—resulting in a 20-item Czech version.

Each item begins with the stem, “I take part in PE ….” Sample items for each subscale include: Intrinsic Motivation (IM; “because PE is fun”), Identified Regulation (IDE; “because it is important for me to do well in PE”), Introjected Regulation (INT; “because I’ll feel bad about myself if I didn't”), External Regulation (EXT; “because I’ll get into trouble if I don't”), and Amotivation (AMO; “but I really don't know why”). Response formats were based on a 7-point Likert-type scale, ranging from 1 (strongly disagree) to 7 (strongly agree).

#### Psychological needs satisfaction in PE

The degree to which students' basic psychological needs were satisfied in PE was assessed using a 15-item measure adapted from prior studies, with strong evidence of reliability and validity in PE settings (23). Responses were given on a 5-point Likert-type scale from 1 (strongly disagree) to 5 (strongly agree).

Autonomy was assessed with five items, each prefaced by the stem, “In this PE class …,” capturing students' sense of choice and control (24). Sample items include, “I have some choice in what I want to do” and “I have a say regarding what skills I want to practice.” Internal consistency for this scale in the current sample was acceptable (*α* = .84). Relatedness was measured using the acceptance subscale from the Need for Relatedness Scale (25). Items were modified to specifically reflect the PE context (e.g., “With the other students in my PE class, I feel …”) and included descriptors such as “close,” “valued,” and “supported.” Internal consistency for this scale was excellent (*α* = .90). Competence was measured using a modified version of the Perceived Competence subscale from the Intrinsic Motivation Inventory (26). Items were adapted to fit the PE setting (e.g., “I am pretty skilled at PE”). The scale demonstrated high reliability (*α* = .89).

#### PE engagement

The level of academic engagement in PE classes was measured using four items with response options ranging from 1 (strongly disagree) to 5 (strongly agree). Sample items include: “I actively prepare the yard and training equipment”, or “I attend PE classes fully and on time”. The set of items was used in a prior study on PE engagement (27), and internal consistency for this scale in the current sample was adequate (*α* = .81).

#### Analytic strategy

We used a multifaceted analytic approach to ascertain the scale's psychometric properties. First, basic descriptive analysis of the PLOC-R items was conducted to examine distributional properties (e.g., location), associations between items (item-total correlation), and hierarchical clustering of respondents within schools (intraclass correlation coefficient – ICC). A 14-day test-retest reliability was estimated for each PLOC-R item and subscale using a reduced sample of participants (*n* = 35). This subsample was recruited separately from the main validation sample due to logistical constraints associated with administering follow-up measurements in school settings. While relatively small, the test–retest sample size is in line with prior psychometric research and was sufficient for estimating item-level and subscale-level temporal stability, providing preliminary evidence of score consistency over time. As a part of the basic analysis, we examined the dimensionality of each PLOC-R subscale using nonparametric Item Response Theory (IRT) approach. Specifically, we used the Mokken's monotone homogeneity (MH) model, which is intended for scaling items on a unidimensional, ordinal scale (28). We calculated Loevinger scalability coefficients [Hi – (29)], for each item within all five PLOC-R subscales using the package “mokken” available in the freeware statistical computing environment R (30). As a rule of thumb, Mokken (28) recommends that in unidimensional scales each item's Hi > 0.3, but a cut-off Hi > 0.5 should be used in order to obtain higher certainty of the MH model fit. In our analysis, items with Hi > 0.5 were flagged as potential sources of misfit and were considered for removal in subsequent dimensionality analyses to improve the psychometric integrity of the scale.

To investigate the scale's dimensionality, we employed a combination of a confirmatory factor analysis (CFA) and several exploratory methods. Specifically, exploratory factor analysis (EFA) was used to identify the most parsimonious fitting factor structure and whose model configuration was theoretically sound. We also used exploratory graph analysis (EGA) to help uncover communities of items based on network analysis (31). In parallel, automated item selection procedure (30) within Mokken scale analysis (MSA) was conducted to identify homogeneous scales from the pool of PLOC items based on scalability criteria. A set of CFAs was carried out to compare both theoretically derived and empirically based models, and multi-group CFA was conducted to test for measurement invariance across gender and grade groups. Model fit in CFA was evaluated using benchmark fit indices including the Comparative Fit Index (CFI), Tucker–Lewis Index (TLI), Root Mean Square Error of Approximation (RMSEA), and Standardized Root Mean Square Residual (SRMR), while *χ*^2^ difference tests and changes in the incremental fit indices were used to ascertain the degree of invariance (32, 33). Both the TLI and CFI have benchmarks close to 1.0 with acceptable fit indicated by values > 0.90 (34), whereas the RMSEA and SRMR should be less than 0.08 in acceptably fitting models (35). Finally, the Bayesian information criterion (BIC) (36) was also used for model fit evaluation (lower values of BIC indicate better model fit). The EFA and CFA models were tested using the Mplus program version 8.11 (37) with robust maximum likelihood estimation to account for departures from (multivariate) normality. Community detection within the EGA was conducted using the R package “EGAnet”, which employed bootstrapped graphical lasso (glasso) network estimation to identify stable clusters of items (31).

Pearson's correlations were computed between the PLOC-R subscale factor scores to test the hypothesis of the simplex-like structure reflecting the self-determination continuum. Finally, to establish the nomological validity of the PLOC-R scores, we examined the associations between the PLOC-R subscales and relevant external measures, including autonomy, relatedness, and competence satisfaction in PE, as well as PE engagement.

## Results

### Basic descriptives for PLOC-R items

Table 2 contains the basic descriptive statistics for the 20 PLOC-R items (correlations for all 20 items can be found in Supplementary S1). Item means ranged from 3.02 (EXT_4) to 4.50 (IM_1) and standard deviations (SD) were fairly consistent (approximately 1.9–2.1), indicating comparable dispersion across items. Skewness and kurtosis values were generally close to zero, suggesting that the distributions of the responses did not deviate markedly from normality.

**Table 2:** PLOC subscale and item descriptives.

Scale/Item	mean	SD	skew	kurt	ICC	mis	item-total r	test-retest^+^	H_i_ - all items	H_i_ - 17 items
Amotivation	3.18	2.09	0.50	−0.97	0.04	3	—	0.90	0.63	0.63
AMO_1 - don't really know why	3.41	2.12	0.37	−1.15	0.04	0	0.65	0.73	0.59	0.59
AMO_2 - don't see why we should have PE	3.05	2.10	0.64	−0.90	0.05	0	0.76	0.82	0.66	0.66
AMO_3 - really feel I'm wasting my time in PE	3.21	2.08	0.52	−0.98	0.04	1	0.76	0.88	0.66	0.66
AMO_4 - can't see what I'm getting out of PE	3.04	2.06	0.63	−0.86	0.03	3	0.71	0.62	0.62	0.62
External Regulation	3.74	2.00	0.20	−0.98	0.01	3	—	0.74	0.36	0.43
EXT_1 - will not get a low grade	4.07	2.07	−0.11	−1.20	0.02	0	0.43	0.59	0.34	0.43
EXT_2 - so that the teacher won't yell at me	3.19	1.95	0.47	−0.91	0.00	0	0.47	0.59	0.37	0.43
EXT_3 - that's the rule	4.68	2.01	−0.45	−0.95	0.01	1	0.43	0.74	0.36	—
EXT_4 - other people say I should*	3.02	1.99	0.60	−0.87	0.01	3	0.45	0.23	0.36	—
Introjected Regulation	3.53	1.98	0.30	−1.04	0.02	3	—	0.78	0.48	0.53
INT_1 - feel bad if the teacher thought that I am not good	3.07	1.97	0.53	−0.93	0.01	0	0.57	0.68	0.47	0.54
INT_2 - feel bad about myself if I did not	3.99	2.03	−0.05	−1.19	0.02	0	0.64	0.61	0.52	0.50
INT_3 - feel bad if the other students thought that I am not good	3.22	1.99	0.44	−1.01	0.01	1	0.59	0.67	0.49	0.55
INT_4 - would bother me if I didn't	3.84	1.92	0.07	−1.03	0.03	3	0.49	0.62	0.42	—
Identified Regulation	3.94	1.90	0.02	−0.99	0.03	3	—	0.90	0.70	0.70
IDE_1 - important to me to improve in drills	3.84	1.89	0.07	−1.00	0.04	0	0.72	0.64	0.66	0.66
IDE_2 - important for me to do well in PE	3.98	1.91	0.00	−1.02	0.04	0	0.78	0.86	0.71	0.71
IDE_3 - important for me to be good in the sports we practice	3.89	1.93	0.09	−1.03	0.03	1	0.81	0.79	0.72	0.72
IDE_4 - important to me to try in PE	4.04	1.86	−0.07	−0.92	0.03	3	0.78	0.82	0.71	0.71
Intrinsic Motivation	4.11	1.88	−0.10	−0.89	0.04	3	—	0.87	0.69	0.69
IM_1 - PE is fun	4.50	1.87	−0.35	−0.82	0.04	0	0.78	0.65	0.71	0.71
IM_2 - enjoy learning new skills	4.15	1.83	−0.11	−0.82	0.04	0	0.67	0.63	0.63	0.63
IM_3 - PE is exciting	3.57	1.89	0.23	−0.94	0.03	1	0.78	0.88	0.72	0.72
IM_4 - PE is enjoyable	4.22	1.92	−0.16	−0.99	0.04	3	0.79	0.67	0.71	0.71

skew, skewness; kurt, kurtosis; ICC, intraclass correlation coefficient; mis, number of missing values; Hi – Scalability coefficient; all items used the stem “I participate in PE (because/but) ..” and abbreviated item content is presented in the table, ^a^item developed by the research team and added to the original PLOC-R scale, ^+^ – test-retest reliability assessed with a sample *n* = 35.

Additionally, Table 2 presents intraclass correlations (ICCs) computed to account for the hierarchical clustering of respondents within schools. The ICC values were uniformly low (ranging from 0.003 to 0.047), indicating that only a minimal proportion of variance in item responses could be attributed to clustering effects within each school. Therefore, single-level analyses were deemed appropriate and used throughout, as the low between-school variability suggested that multilevel modeling was not necessary. Test–retest reliability (*n* = 35) estimates suggest that item responses were generally stable over time, with most items showing moderate to high coefficients. Item–total correlations (ranging from 0.43 to above 0.88, depending on the subscale) indicate that each item contributes meaningfully to its corresponding factor. Moreover, the Loevinger scalability coefficients (Hi) derived from MSA provide evidence of satisfactory scalability, as most items met or exceeded the desired threshold (Hi ≥ 0.50), reinforcing the unidimensional structure within most of the PLOC subscales.

Several items, however, did not meet the acceptable psychometric benchmark criteria. In particular, item EXT_4 showed notably low test–retest reliability, whereas all items measuring external regulation (EXT_1 to EXT_4) together with INT_4 (i.e., “because it would bother me if I didn't”) displayed suboptimal item–total correlations and lower scalability coefficients. Removal of INT_4 enhanced the properties of introjected regulation, as scalability coefficients for individual items, as well as for the whole subscale, were above the required threshold of 0.5. Furthermore, the subsequent removal of EXT_3 (i.e., “because that's the rule”) and EXT_4 (i.e., “because other people say I should”) also led to an overall improvement in the psychometric properties of the external regulation subscale; however, scalability coefficients for both remaining items still did not reach the desired threshold.

Bivariate relationships among the items (provided as Supplementary Material) were in an expected direction (e.g., intrinsic motivation items correlated positively with interjected regulation items and negatively with amotivation items) and within reasonable range, laying a solid foundation for further analyses on the questionnaire's dimensionality.

### Dimensionality of PLOC-R

We examined several different model specifications for PLOC-R dimensionality, based on both theory-driven and empirically-driven perspectives. Table 3 summarizes how the PLOC-R items were grouped across latent factors in the model configurations.

**Table 3:** PLOC item clustering for tested models.

Motivation type	Scale	Item	M1 Theoretical 5-factor model	M2 Theoretical 3-factor model	M3 3-factor model based on EFA	M4 3-factor model based on EGA	M5 3-factor model based on MSA	M6 Theoretical 5-factor model, revised
Amotivation	Amotivation	amo_1	1	1	1	1	1	1
		amo_2	1	1	1	1	1	1
		AMO_3	1	1	1	1	1	1
		AMO_4	1	1	1	1	1	1
Controlled motivation	External regulation	EXT_1	2	2	2	2	2	2
		EXT_2	2	2	2	2	2	2
		EXT_3	2	2	2	1	1	Excl.
		EXT_4	2	2	1	1	1	Excl.
	Introjected regulation	INT_1	3	2	2	2	3	3
		INT_2	3	2	2	2	3	3
		INT_3	3	2	2	2	3	3
		INT_4	3	2	2	3	3	Excl.
Autonomous motivation	Identified regulation	IDE_1	4	3	3	3	3	4
		IDE_2	4	3	3	3	3	4
		IDE_3	4	3	3	3	3	4
		IDE_4	4	3	3	3	3	4
	Intrinsic motivation	IM_1	5	3	3	3	3	5
		IM_2	5	3	3	3	3	5
		IM_3	5	3	3	3	3	5
		IM_4	5	3	3	3	3	5

EFA, exploratory factor analysis; EGA, exploratory graph analysis; MSA, Mokken scale analysis; Excl., item was excluded.

In the first step, we examined the original theoretically postulated 5-factor model (M1), which showed a less than optimal fit to the data (TLI = 0.893, CFI = 0.873, RMSEA = 0.077, SRMR = 0.097). Next, we tested another theoretically plausible model (M2), which distinguished autonomous motivation, controlled motivation, and amotivation. This model also did not provide a good fit to the data (see Table 4 for fit indices). At this point, we aimed to search for alternative models using exploratory approaches including EFA, EGA, and MSA.

**Table 4:** CFA fit indices for different models of PLOC items.

Model	*χ*^2^ (df)	CFI	TLI	RMSEA	SRMR	AIC	BIC
M1 - Theoretical 5-factor model	2,987.7 (160)	0.893	0.873	0.077	0.970	216,675.9	217,095.5
M2 - Theoretical 3-factor model	5,720.6 (167)	0.789	0.760	0.106	0.162	220,033.9	220,411.6
M3 - 3-factor model based on EFA	5,037.6 (167)	0,815	0,790	0,099	0,139	219,220.8	219,598.5
M4 - 3-factor model based on EGA	3,874.2 (167)	0,859	0,840	0,086	0.102	217,826.7	218,204.4
M5 - 3-factor model based on MSA	5,155.8 (167)	0.811	0.785	0.100	0.103	219,514.1	219,891.8
M6 - Theoretical 5-factors, revised	1,377.4 (109)	0.944	0.930	0.063	0.054	181,912.7	182,278.4

df, degrees of freedom; CFI, comparative fit index; TLI, Trucker-Lewis index; RMSEA, root mean square error of approximation; SRMR, standardized root mean square residual; AIC, Akaike information criterion; BIC - Bayesian information criterion.

A detailed description of results for EFA (table of fit indices for 1-6 factor EFA and factor loadings for the selected model), EGA (figure of bootstrapped EGA network and clustering) and MSA (table with identified scales for increasing scalability coefficient) are provided in Supplementary S2–S5. The most parsimonious EFA model with acceptable model fit was a three-factor model. The MSA monotone homogeneity model with Hi cutoff of 0.3, also identified 3 scales. The EGA also supported a 3-dimensional structure. Specifically, the network analysis revealed three distinct communities of items, corresponding to the three latent dimensions, as indicated by the number of nodes (items) and edges (relationships between items). Taken together, the exploratory approaches all identified a 3-dimensional structure of PLOC-R items, with item clustering mimicking the theoretically plausible 3-factor model M2.

PLOC-R structures suggested by exploratory approaches (model M3, M4 and M5) were subsequently compared using CFA in terms of relative model fit. Both AIC and BIC suggested that the EGA-based CFA model (M4) is preferred over the other two models under scrutiny (M3 and M5). All absolute fit indices for model M4 (see Table 4) were, however, either below (CFI, TLI) or above (RMSEA, SRMS) acceptable levels for a good fitting model. Moreover, both the absolute and relative fit indices for this model were slightly worse than fit indices for the originally postulated 5-factor model M1.

Finally, based on preliminary item analyses that identified three problematic items, we refined the originally postulated 5-factor model by excluding 3 items, namely EXT_3, EXT_4 and INT_4.This revised 5-factor model (M6), now incorporating only 17 items, demonstrated improved fit indices compared to the original M1, indicating that the removal of these items enhanced the validity of the PLOC-R's dimensionality interpretation. Given that the absolute fit indices met the criteria for a well-fitting model, we selected model M6 as the final model for interpretation and further analyses.

### Internal structure, reliability and nomological validity of PLOC subscales

The standardized factor loadings for the final model M6 (listed in Supplementary S6) varied from a low of .56 for EXT_1, to a high of. 86 for IDE_3 (average *λ* = .78). The strongest inter-factor correlations (Table 5, upper part) were observed between scales measuring IM and IDE (r = 0.89) suggesting substantial overlap of information captured by these factors. Nevertheless, the overall pattern of factor correlations provided support for a simplex ordering of the PLOC-R subscales, in which constructs that are closer together on the SDT continuum exhibit stronger correlations than those that are further apart. For instance, the AMO subscale showed a high positive correlation with EXT, a low negative correlation with INT, and a strong negative correlation with IDE and IM subscales. The estimates of internal consistency based on the common factor model (*ω*) for each PLOC-R subscale ranged from a low of 0.58 for EXT to a high of 0.90 for IDE. An *ω* = 0.58 indicates that the internal consistency of the EXT subscale falls below commonly accepted thresholds for reliability. As such, the subscale may be more suitable for screening purposes, and extending it with additional well-performing items would likely improve its reliability, as internal consistency tends to increase with test length.

**Table 5:** Correlations and mcDonald's omega for study variables.

Variable	1	2	3	4	5	*ω*
Motivation regulations in PE						
1. Amotivation (AMO)	1					0.87
2. External Regulation (EXT)	0.30 (0.25, 0.35)	1				0.58
3. Introjected Regulation (INT)	−0.17 (−0.11, −0.22)	0.75 (0.70, 0.80)	1			0.78
4. Identified Regulation (IDE)	−0.66 (−0.63, −0.69)	0.13 (0.08, 0.19)	0.64 (0.60, 0.68)	1		0.90
5. Intrinsic Motivation (IM)	−0.76 (−0.73, −0.78)	−0.11 (−0.05, −0.17)	0.35 (0.30, 0.40)	0.88 (0.86, 0.90)	1	0.89
External measures						
Autonomy satisfaction	−0.39 (−0.34, −0.44)	−0.17 (−0.12, −0.22)	0.07 (0.02, 0.12)	0.39 (0.34, 0.43)	0.54 (0.50, 0.59)	0.84
Relatedness satisfaction	−0.26 (−0.21, −0.30)	−0.03 (0.02, −0.09)	0.13 (0.08, 0.18)	0.33 (0.28, 0.37)	0.38 (0.34, 0.42)	0.90
Competence satisfaction	−0.49 (−0.45, −0.52)	−0.24 (−0.19, −0.29)	0.05 (0.00, 0.10)	0.48 (0.45, 0.52)	0.61 (0.58, 0.65)	0.89
PE engagement	−0.51 (−0.48, −0.55)	0.06 (0.01, 0.11)	0.32 (0.27, 0.37)	0.57 (0.54, 0.60)	0.56 (0.52, 0.59)	0.81

Correlations of factor score estimates are displayed, correlations larger than 0.05 in absolute values are statistically significant at *α* = 0.05, 95% confidence intervals are provided in brackets.

The correlations between the PLOC-R subscales and external variables (Table 5, lower part) revealed a clear support for the theoretical formulations in SDT. In general, autonomous forms of motivation—namely, IDE and IM—show moderate to strong positive correlations (r ≥ 0.33) with a satisfaction of all three psychological needs (autonomy, relatedness, and competence), and PE engagement. Additionally, INT was positively associated with all external measures; however, its effect sizes were lower than those for the autonomous forms of motivation. Conversely, AMO showed moderate negative correlations (ranging from −0.26 to −0.51) with all external measures. Finally, EXT exhibited weak correlations—with slightly negative associations for autonomy and competence satisfaction and minimal associations with relatedness satisfaction and engagement. Overall, the results support the hypothesized latent factor structure using a reduced set of 17 items and reinforce the PLOC-R's construct and nomological validity in our sample.

### Measurement invariance

We next conducted a multi-group analysis to test for measurement invariance of the hypothesized PLOC-R model across gender (males vs. females), and grade (1st and 2nd vs. 3rd and 4th graders). Table 6 contains the fit indices for the full set of invariance models.

**Table 6:** Measurement invariance model fit indices.

Model	χ^2^ (df)	CFI	TLI	RMSEA	SRMR	Δ p	Δ CFI	Δ TLI	Δ RMSEA	Δ SRMR
Gender: Males (*n* = 1,348) vs. Females (*n* = 1,558)										
Configural	1,436.33 (218)	0.945	0.931	0.062	0.053					
Metric	1,472.44 (230)	0.944	0.934	0.061	0.054	0.044	0.001	−0.003	0.001	−0.001
Scalar	1,665.33 (242)	0.936	0.928	0.064	0.057	<0.001	0.008	0.006	−0.003	−0.003
Strict	1,675.69 (259)	0.936	0.933	0.061	0.059	0.026	0.000	−0.005	0.003	−0.002
Grade: 1st, 2nd graders (*n* = 1,475) vs. 3rd, 4th graders (*n* = 1,438)										
Configural	1,494.60 (218)	0.943	0.929	0.063	0.055					
Metric	1,528.20 (230)	0.942	0.932	0.062	0.055	0.203	0.001	−0.003	0.001	0.000
Scalar	1,582.96 (242)	0.940	0.933	0.061	0.055	<0.001	0.002	−0.001	0.001	0.000
Strict	1,577.04 (259)	0.941	0.938	0.059	0.056	0.642	−0.001	−0.005	0.002	−0.001

df, degrees of freedom; CFI, comparative fit index; TLI, Trucker-Lewis index; RMSEA, root mean square error of approximation; SRMR, standardized root mean square residual; *Δ* - difference between two adjacent models, where more restricted model is always compared to a less restricted model (e.g., metric versus configural; scalar versus metric and strict versus scalar), grade levels were collapsed into two groups (1st–2nd vs. 3rd–4th grade) based on preliminary analysis supporting strict measurement invariance and to ensure sufficient group sizes for multi-group testing.

For both grouping variables, the baseline configural model positing the same configuration of factors across the groups exhibited a good fit with the data. The imposition of equality constraints on factor loadings in the model positing metric invariance resulted in a non-significant chi-square difference for grade [*Δχ*^2^(12) = 15.7, *p* = 0.203], and only a marginally significant difference for gender [*Δχ*^2^(12) = 21.5, *p* = 0.044], with negligible changes in the incremental fit indices. Although the scalar model with equal item intercepts produced statistically significant chi-square differences for both gender and grade (*p* < 0.001) – likely due to the large sample size—the corresponding changes in the incremental fit indices remained trivial. The strict invariance model further confirmed invariance of item residuals for grade, as evidenced by a non-significant chi-square difference [*Δχ*^2^(17) = 14.3, *p* = 0.642]. Overall, despite statistically significant chi-square differences in some cases, the minimal changes in CFI, TLI, RMSEA, and SRMR across all models (< 0.01) indicate that a strict level of invariance is achieved for both gender and grade groups.

## Discussion

The present study set out to adapt and validate the PLOC-R scale assessing PE motivation in a Czech high school sample. In line with expectations from SDT, the PLOC-R's five-factor structure was largely confirmed, though initial model fit was unsatisfactory until several problematic items were identified and removed.

### Factor structure and model fit

The original five-factor model with 20 items did not achieve an acceptable fit in the Czech sample (e.g., CFI < 0.90) suggesting that some questionnaire items were not well-aligned with the intended latent factors. We also tested a coarser, three-factor model (collapsing intrinsic + identified, and introjected + external, plus amotivation) to see if students might only distinguish broad motivation categories and not make finer distinctions between different forms of motivation. This three-factor model fit the data even more poorly, indicating that important information regarding motivational styles would be lost by merging the regulation types. Thus, the fine-grained SDT framework positing five forms of behavioral regulations remained conceptually necessary—even if some items did not perform as expected.

To diagnose the misfit, we turned to exploratory techniques (EFA, EGA, and MSA), which all converged on a three-factor clustering of items, roughly corresponding to autonomous motivation, controlled motivation, and amotivation, This raised the question of whether the theoretical five factors are truly distinct in practice (4), or whether certain subscales are empirically hard to distinguish. However, when we compared various models via CFA, the original five-factor model still showed better relative fit than the exploratory three-factor solutions. We interpreted this to mean that the five hypothesized constructs are psychometrically sound, but some items may be causing cross-loadings or noise that obscure a purer factor structure. By identifying and removing these problematic items (discussed below), we were able to recover a well-fitting five-factor solution. Notably, our refined model's fit indices are very much in line with those reported in other countries after similar refinements (14, 16), which mirrors the global pattern that the PLOC-R can be successfully adapted, provided that any culture-specific problematic items are addressed.

### Item refinement

The decision to remove three specific items (EXT_3, EXT_4, and INT_4) was driven by empirical criteria and proved crucial for achieving a valid measurement model. In practical terms, students’ responses to these statements did not reliably track with their corresponding subscale, suggesting potential issues in translation or interpretation. For instance, one of the dropped introjected items was “Because I want the PE teacher to think I am a good student,” which in our analyses tended to correlate similarly with external and introjected regulation. This finding mirrors exactly what Teixeira et al. (16) observed in their study of Portuguese youth—a particular item cross-loaded onto the wrong factor, likely because seeking teacher approval can be perceived as an external incentive by some students. In our sample, a similar ambiguous interpretation of the item content may have occurred, blurring the line between doing PE to please the teacher (introjected pride/shame) vs. doing it to acquiesce to teacher pressure (external control). Removing such items improved the factorial clarity of the scale.

Likewise, two EXT items were eliminated, which is consistent with prior adaptations of PLOC-R. Items for the EXT subscale often pose challenges because extrinsic motives are heterogeneous—e.g., some students focus on grades or rewards, others on avoiding punishment—making it harder for a single set of items to form a coherent dimension. In the Chinese version of PLOC-R (17), several external/introjected items had to be dropped for similar reasons: they did not function equivalently following translation and undermined model fit. Our Czech adaptation followed an almost identical pattern (three items removed, 17-item final scale), underlining how culture-specific issues can affect certain PLOC-R items. What works well in one language may not directly translate—subtle wording differences can shift an item's meaning enough that it no longer aligns with its intended construct. It is also notable that no PLOC-R language adaptation to date has required that a significant number of items be removed—rather, at most, 2–3 items are removed or replaced. This consistency attests to the theoretical consistency of PLOC-R's original design.

### Internal consistency of subscales

The internal consistency of four out of five PLOC-R subscales validated with the Czech sample was acceptable to excellent, providing further evidence that the translated items reliably capture the underlying motivational constructs. McDonald's omega values for IDE and IM were especially high (*ω* = 0.90), and INT was in a respectable range (*ω* = 0.78). These figures are comparable to or slightly higher than those reported in other language validation studies [e.g. (38)]. For instance, the original developers (13) found Cronbach's *α* > .70 for all subscales in English, and a large Spanish study (14) likewise had *α* > .70 on most subscales. The recent German PLOC-R [which even added an extra subscale (15),] reported Cronbach's *α* from 0.81 (external) up to 0.93 (integrated). Our results generally fall in line with this literature, confirming that the Czech PLOC-R scales are internally reliable—with one important exception.

The EXT subscale in our study showed a relatively lower internal consistency (*ω* = 0.58), which is below conventional benchmarks. This is not entirely surprising given that we had to shorten this subscale to only two items. Fewer items inherently limit reliability, and the two remaining items may capture somewhat disparate aspects of external motivation. Other studies have also noted lower reliability for external regulation, even with more items. For example, Teixeira et al. (16) found composite reliability for EXT just under 0.70 in one of their samples (though still above 0.60). They considered this acceptable for a factor with few items and pointed out that a similar issue was observed in a Hong Kong sample (39). Thus, while the value *ω* = 0.58 undermines scale integrity, it reflects a broader pattern where the construct of external regulation is challenging to measure in youthful samples with high consistency. It may be worthwhile in future to add more items and obtain a richer picture of what is meant by external regulation or refine the current phrasing to strengthen the subscale's reliability. Given the low internal consistency, the EXT subscale in its current form is more appropriate for screening group-level differences than for precise assessment of within- or between-individual variation. Encouragingly, all other subscales in the Czech PLOCQ demonstrated more than adequate reliability, suggesting that aside from external regulation, the instrument yields stable and consistent scores for the different motivation types.

### Correlations and nomological validity

Evidence for the PLOC-R's construct validity in our sample comes not only from factor structure, but also from the pattern of inter-factor correlations and relations with external criteria. As expected, the five motivation subscales exhibited a simplex-like correlation structure consistent with SDT's continuum (40). Motivational regulations that are conceptually adjacent (e.g., identified and intrinsic) demonstrated strong positive inter-correlations, whereas those at opposite ends (intrinsic vs. amotivation) had negative correlations. For example, IM correlated very highly with IDE (r ≈ 0.89) in our data. This is a common occurrence in PLOC studies: intrinsic and identified forms of motivation often overlap substantially, as students who truly enjoy PE also tend to recognize its personal value. Studies conducted in Germany and Portugal (15, 16) also reported that these two subscales were not easily distinguishable (sometimes failing strict discriminant validity tests). These results may suggest that high school students do not always differentiate between the more specific forms of motivational regulations (e.g., intrinsic vs. identified) and instead may categorize motivation in broader terms—such as autonomous vs. controlled, or motivated vs. amotivated. By contrast, AMO in our study related negatively to self-determined forms (*r* = –0.75 with IM) and positively to EXT (*r* = 0.30), again aligning with the theorized continuum and prior research (12).

Beyond internal relationships, we also examined nomological validity by correlating PLOC-R scores with external measures (basic need satisfaction and PE engagement). The correlations followed theoretically meaningful directions, providing further validation evidence. For instance, the pattern of association between IM with engagement and likewise with autonomy satisfaction, indicated that students who are internally motivated tend to be more involved in class and feel more autonomous. Conversely, AMO was negatively associated with the beneficial outcomes, suggesting that amotivated students are the least engaged and feel the least satisfied in PE. Prior studies have also shown that intrinsically motivated students report greater enjoyment, effort, and preference for challenge, whereas amotivated or externally driven students report lower interest and participation (4). By demonstrating these same associations in the Czech setting, we bolster the argument that the PLOC-R is not only structurally sound but also meaningfully connected to important educational predictors and outcomes (i.e., it has predictive and convergent validity).

### Measurement invariance

The multi-group CFA results indicated that the PLOC-R factor structure and item parameters were equivalent for boys and girls as well as for younger vs. older students, demonstrating configural, metric, scalar, and even strict invariance. These findings are in line with prior cross-cultural studies of the PLOC and PLOC-R (12, 38). The fact that the Czech PLOC-R also demonstrated invariance suggests that the instrument measures motivational regulations equivalently across gender and age subgroups, implying that any differences in scores between males and females or across grade levels reflect true differences in motivation rather than measurement bias. Researchers and practitioners can thus be confident in comparing Czech PLOC-R scores across genders and late adolescent age groups.

### Implications for research and practice

Validating the Czech version of the PLOC-R fills a gap in the toolkit for researchers and educators who conduct research on motivation in this region. Having a sound PLOC-R means scholars can confidently include Czech samples in cross-cultural studies of PE motivation. Until now, comparisons of student motivation across countries could not easily include Czech data. With the 17-item Czech PLOC-R, it becomes feasible to examine research questions like “Do Czech students have similar motivational profiles to their peers in other European countries?” or “How do cultural and educational differences influence the balance of autonomous vs. controlled motivation?” For example, researchers could replicate studies such as Wang et al. (12) by comparing Czech students’ PLOC-R scores with those from more individualistic or collectivistic cultures. Our results suggest the measure would behave equivalently, given the strong cross-cultural evidence of factorial invariance.

For PE practitioners, the PLOC-R offers a practical diagnostic tool. Teachers and school psychologists can administer the Czech PLOC-R to obtain a snapshot of their respective classes' motivational climate. The results might identify, for example, a class that is high in introjected and external motivation but low in intrinsic motivation. This could alert the teacher that students participate due to feelings of guilt or obligation rather than genuine interest, which might lead to burnout or dropout. With this information, targeted strategies (like increasing task variety, offering more choice, or emphasizing fun and personal relevance) could shift the motivational climate toward more self-determined reasons. The availability of a validated Czech PLOC-R allows research and applied efforts to be grounded in a well-established theoretical measure, bringing Czech PE closer to the standard in countries that have long been using motivation questionnaires to inform practice.

Looking ahead, one intriguing implication of the strong intrinsic–identified correlation is whether, in older adolescents or young adults, these two forms might merge unless integrated regulation is introduced. Recent work in German high schools (15) showed that a six-factor model (including integrated regulation) can capture additional regulatory experiences at the high end of self-determination. For Czech late-teen populations, adding integrated motivation items could be a worthwhile direction to explore. Our current validation sets the stage for that extension by confirming the core five factors first. Overall, the Czech PLOC-R appears valid and reliable to assess PE motivation, paving the way for richer investigations into how and why students become autonomously motivated in PE.

### Limitations and future research

Several limitations should be acknowledged. First, the sample consisted of Czech high school students from a limited geographic region, restricting the generalizability of findings. Second, the study's cross-sectional design does not allow for conclusions about changes in motivation over time. Longitudinal research is needed to assess the stability of motivation and whether students shift along the self-determination continuum.

The removal of three items highlights that some constructs might be perceived differently in Czech educational settings. Future work should consider conducting qualitative research—such as in-depth cognitive interviews or focus groups—particularly focused on the external regulation subscale, to better understand how high school students conceptualize externally regulated motivation and to inform the development of refined or new items that enhance the subscale's clarity and psychometric performance. Additionally, the external regulation subscale exhibited lower internal consistency, likely due to having only two retained items. This mirrors similar issues in other PLOC-R adaptations, suggesting that a third (and a fourth) item may be needed to improve measurement precision.

To further strengthen the Czech PLOC-R's validity, future studies should investigate predictive validity, testing whether motivation profiles measured with the PLOC-R predict long-term engagement in PA. Expanding validity assessments—such as comparing PLOC-R scores with teacher evaluations or behavioral observations—would confirm its real-world applicability. Addressing these areas can refine the instrument and enhance its usability for cross-cultural comparisons and practical applications in PE.

## Conclusion

The present study validated the Czech version of the PLOC-R scale in a large sample of high school students. After the removal of three problematic items, the revised 17-item scale demonstrated an acceptable five-factor structure consistent with SDT. The refined model showed good internal consistency for most subscales, with the exception of external regulation, which remained relatively low due to the reduced number of items. The final model exhibited a theoretically expected correlation structure and strong evidence of construct validity through associations with psychological need satisfaction and PE engagement. Importantly, measurement invariance was confirmed across gender and grade levels, supporting the use of the Czech PLOC-R for group comparisons in adolescent populations. These findings establish a psychometrically sound instrument for assessing PE motivation among Czech youth.

## Data Availability

The raw data supporting the conclusions of this article can be found here: https://cunicz-my.sharepoint.com/:x:/g/personal/86956275_cuni_cz/EUixUPEHt9dEpr4FvvSHEYgB9PlMjgBGFXV1h0KlHFrjTg?e=hx7krx.
