# Supplementary material for: Psychometric validation of the Czech PLOC-R in high-school physical education
Source: Front Sports Act Living. 2025 Sep 30;7:1629138. doi: 10.3389/fspor.2025.1629138 (PMC12518339; doi:10.3389/fspor.2025.1629138)
Supplement: Supplementary file 1 [file Datasheet1.docx]

**Supplement 1: Correlation matrix of PLOC items.**

|  | AMO_1 | AMO_2 | AMO_3 | AMO_4 | EXT_1 | EXT_2 | EXT_3 | EXT_4 | INT_1 | INT_2 | INT_3 | INT_4 | IDE_1 | IDE_2 | IDE_3 | IDE_4 | IM_1 | IM_2 | IM_3 |
| --- | --- | --- | --- | --- | --- | --- | --- | --- | --- | --- | --- | --- | --- | --- | --- | --- | --- | --- | --- |
| AMO_1 | 1 |  |  |  |  |  |  |  |  |  |  |  |  |  |  |  |  |  |  |
| AMO_2 | .600^**^ | 1 |  |  |  |  |  |  |  |  |  |  |  |  |  |  |  |  |  |
| AMO_3 | .578^**^ | .714^**^ | 1 |  |  |  |  |  |  |  |  |  |  |  |  |  |  |  |  |
| AMO_4 | .551^**^ | .626^**^ | .662^**^ | 1 |  |  |  |  |  |  |  |  |  |  |  |  |  |  |  |
| EXT_1 | .183^**^ | .168^**^ | .157^**^ | .148^**^ | 1 |  |  |  |  |  |  |  |  |  |  |  |  |  |  |
| EXT_2 | .185^**^ | .168^**^ | .150^**^ | .151^**^ | .402^**^ | 1 |  |  |  |  |  |  |  |  |  |  |  |  |  |
| EXT_3 | .308^**^ | .264^**^ | .337^**^ | .274^**^ | .306^**^ | .275^**^ | 1 |  |  |  |  |  |  |  |  |  |  |  |  |
| EXT_4 | .377^**^ | .406^**^ | .445^**^ | .460^**^ | .252^**^ | .363^**^ | .394^**^ | 1 |  |  |  |  |  |  |  |  |  |  |  |
| INT_1 | -.008 | -.034 | -.064^**^ | -.021 | .363^**^ | .410^**^ | .036^*^ | .141^**^ | 1 |  |  |  |  |  |  |  |  |  |  |
| INT_2 | -.187^**^ | -.228^**^ | -.267^**^ | -.197^**^ | .224^**^ | .331^**^ | .044^*^ | .017 | .457^**^ | 1 |  |  |  |  |  |  |  |  |  |
| INT_3 | -.018 | -.030 | -.045^*^ | -.023 | .265^**^ | .427^**^ | .101^**^ | .192^**^ | .583^**^ | .490^**^ | 1 |  |  |  |  |  |  |  |  |
| INT_4 | -.330^**^ | -.374^**^ | -.426^**^ | -.340^**^ | .064^**^ | .155^**^ | -.108^**^ | -.138^**^ | .315^**^ | .555^**^ | .335^**^ | 1 |  |  |  |  |  |  |  |
| IDE_1 | -.400^**^ | -.435^**^ | -.480^**^ | -.411^**^ | .034 | 0.028 | -.246^**^ | -.230^**^ | .306^**^ | .396^**^ | .241^**^ | .521^**^ | 1 |  |  |  |  |  |  |
| IDE_2 | -.387^**^ | -.421^**^ | -.468^**^ | -.390^**^ | .080^**^ | .127^**^ | -.171^**^ | -.200^**^ | .368^**^ | .558^**^ | .363^**^ | .582^**^ | .643^**^ | 1 |  |  |  |  |  |
| IDE_3 | -.403^**^ | -.452^**^ | -.515^**^ | -.439^**^ | .029 | .069^**^ | -.216^**^ | -.246^**^ | .325^**^ | .476^**^ | .344^**^ | .587^**^ | .665^**^ | .742^**^ | 1 |  |  |  |  |
| IDE_4 | -.400^**^ | -.425^**^ | -.497^**^ | -.408^**^ | .054^**^ | .104^**^ | -.165^**^ | -.184^**^ | .324^**^ | .520^**^ | .335^**^ | .681^**^ | .649^**^ | .702^**^ | .725^**^ | 1 |  |  |  |
| IM_1 | -.482^**^ | -.542^**^ | -.597^**^ | -.492^**^ | -.114^**^ | -.134^**^ | -.293^**^ | -.369^**^ | .132^**^ | .287^**^ | .091^**^ | .458^**^ | .613^**^ | .550^**^ | .582^**^ | .544^**^ | 1 |  |  |
| IM_2 | -.410^**^ | -.434^**^ | -.475^**^ | -.407^**^ | .013 | .031 | -.211^**^ | -.240^**^ | .232^**^ | .423^**^ | .196^**^ | .510^**^ | .660^**^ | .695^**^ | .645^**^ | .622^**^ | .593^**^ | 1 |  |
| IM_3 | -.412^**^ | -.456^**^ | -.525^**^ | -.441^**^ | -.041^*^ | 0.005 | -.267^**^ | -.271^**^ | .209^**^ | .332^**^ | .195^**^ | .504^**^ | .620^**^ | .620^**^ | .682^**^ | .609^**^ | .694^**^ | .633^**^ | 1 |
| IM_4 | -.467^**^ | -.522^**^ | -.590^**^ | -.491^**^ | -.101^**^ | -.083^**^ | -.272^**^ | -.325^**^ | .128^**^ | .306^**^ | .104^**^ | .529^**^ | .578^**^ | .555^**^ | .611^**^ | .620^**^ | .752^**^ | .600^**^ | .716^**^ |

**Supplement 2: Fit indices for EFA**

| Model | Chi-Square | df | p | RMSEA | CFI | TLI | SRMR |
| --- | --- | --- | --- | --- | --- | --- | --- |
| 1-factor | 8528.8 | 170 | <0.001 | 0.129 | 0.683 | 0.646 | 0.127 |
| 2-factor | 2892.2 | 151 | <0.001 | 0.078 | 0.896 | 0.869 | 0.038 |
| **3-factor** | **1612.8** | **133** | <0.001 | **0.061** | **0.944** | **0.920** | **0.026** |
| 4-factor | 1130.1 | 116 | <0.001 | 0.054 | 0.962 | 0.937 | 0.022 |
| 5-factor | 832.4 | 100 | <0.001 | 0.05 | 0.972 | 0.947 | 0.018 |
| 6-factor | 524.1 | 85 | <0.001 | 0.042 | 0.983 | 0.963 | 0.012 |

**Supplement 3: Standardized factor loadings of 3-factor EFA model. Factor loadings <0.3 (in absolute value) are omitted**

|  | Factor 1 | Factor 2 | Factor 3 |
| --- | --- | --- | --- |
| amo_1 | 0.643 |  |  |
| amo_2 | 0.859 |  |  |
| amo_3 | 0.794 |  |  |
| amo_4 | 0.778 |  |  |
| ext_1 |  | 0.464 |  |
| ext_2 |  | 0.615 |  |
| ext_3 |  | 0.304 |  |
| ext_4 | 0.438 | 0.320 |  |
| int_1 |  | 0.615 |  |
| int_2 |  | 0.586 |  |
| int_3 |  | 0.670 |  |
| int_4 |  |  | 0.547 |
| ide_1 |  |  | 0.813 |
| ide_2 |  |  | 0.771 |
| ide_3 |  |  | 0.812 |
| ide_4 |  |  | 0.751 |
| im_1 |  |  | 0.784 |
| im_2 |  |  | 0.841 |
| im_3 |  |  | 0.942 |
| im_4 |  |  | 0.828 |

**Supplement 4: Figure of estimated EGA network.**


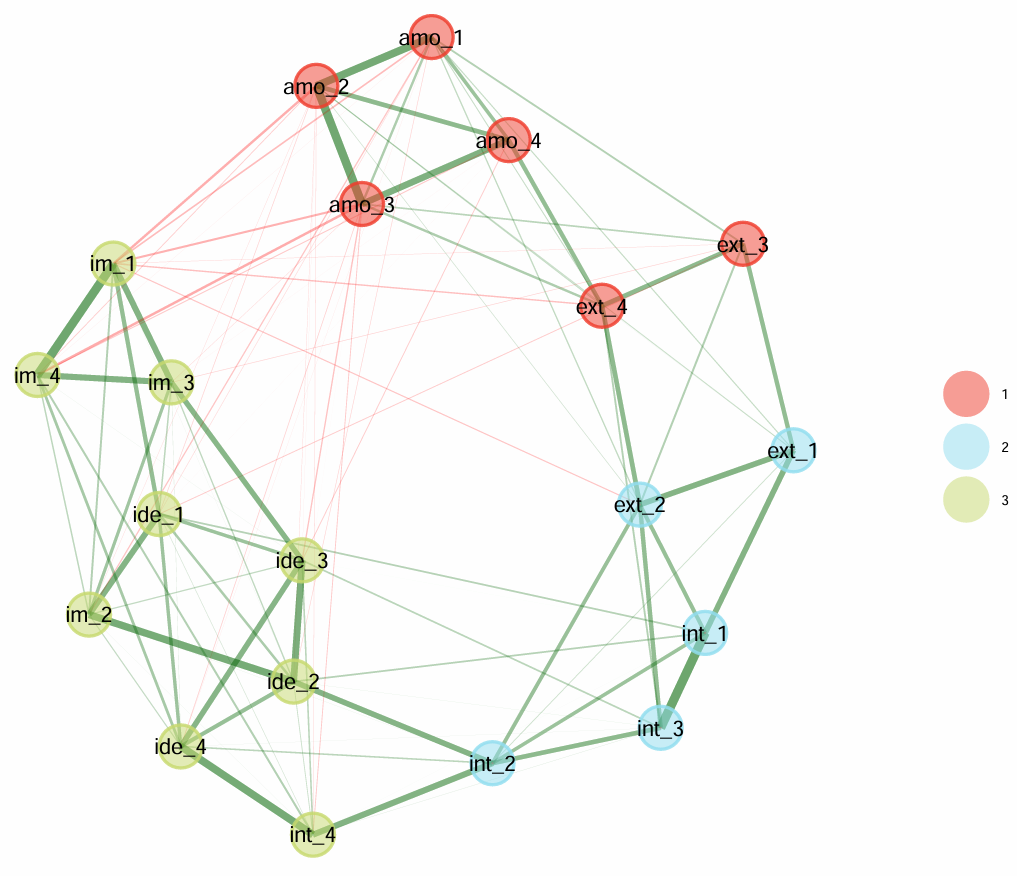


**Supplement 5: Scales as per increasing level of Loevinger’s scalability coefficient (Hi)**

|  | Hi = 0.3 | Hi = 0.35 | Hi = 0.4 | Hi = 0.45 |
| --- | --- | --- | --- | --- |
| amo_1 | 2 | 2 | 2 | 2 |
| amo_2 | 2 | 2 | 2 | 2 |
| amo_3 | 2 | 2 | 2 | 2 |
| amo_4 | 2 | 2 | 2 | 2 |
| ext_1 | 3 | 3 | 0 | 0 |
| ext_2 | 3 | 3 | 3 | 0 |
| ext_3 | 2 | 2 | 0 | 0 |
| ext_4 | 2 | 2 | 2 | 0 |
| int_1 | 1 | 3 | 3 | 3 |
| int_2 | 1 | 1 | 1 | 3 |
| int_3 | 1 | 3 | 3 | 3 |
| int_4 | 1 | 1 | 1 | 1 |
| ide_1 | 1 | 1 | 1 | 1 |
| ide_2 | 1 | 1 | 1 | 1 |
| ide_3 | 1 | 1 | 1 | 1 |
| ide_4 | 1 | 1 | 1 | 1 |
| im_1 | 1 | 1 | 1 | 1 |
| im_2 | 1 | 1 | 1 | 1 |
| im_3 | 1 | 1 | 1 | 1 |
| im_4 | 1 | 1 | 1 | 1 |

**Supplement 6: Standardized factor loadings for the final CFA model using 17 PLOC items.**

|  | AMO | EXT | INT | IDE | IM |
| --- | --- | --- | --- | --- | --- |
| amo_1 | 0.707 |  |  |  |  |
| amo_2 | 0.824 |  |  |  |  |
| amo_3 | 0.861 |  |  |  |  |
| amo_4 | 0.765 |  |  |  |  |
| ext_1 |  | 0.560 |  |  |  |
| ext_2 |  | 0.718 |  |  |  |
| ext_3 |  | - |  |  |  |
| ext_4 |  | - |  |  |  |
| int_1 |  |  | 0.720 |  |  |
| int_2 |  |  | 0.704 |  |  |
| int_3 |  |  | 0.723 |  |  |
| int_4 |  |  | - |  |  |
| ide_1 |  |  |  | 0.782 |  |
| ide_2 |  |  |  | 0.845 |  |
| ide_3 |  |  |  | 0.863 |  |
| ide_4 |  |  |  | 0.833 |  |
| im_1 |  |  |  |  | 0.83 |
| im_2 |  |  |  |  | 0.762 |
| im_3 |  |  |  |  | 0.836 |
| im_4 |  |  |  |  | 0.847 |
